# CD38 Causes Autophagic Flux Inhibition and Cardiac Dysfunction Through a Transcriptional Inhibition Pathway Under Hypoxia/Ischemia Conditions

**DOI:** 10.3389/fcell.2020.00191

**Published:** 2020-04-17

**Authors:** Xingyue Zhang, Lingfei Li, Qiong Zhang, Qinglin Wei, Jiezhi Lin, Jiezhi Jia, Junhui Zhang, Tiantian Yan, Yanling Lv, Xupin Jiang, Peng Zhang, Huapei Song, Dongxia Zhang, Yuesheng Huang

**Affiliations:** ^1^Institute of Burn Research, Southwest Hospital, Army Medical University, Third Military Medical University, Chongqing, China; ^2^State Key Laboratory of Trauma, Burns and Combined Injury, Southwest Hospital, Army Medical University, Third Military Medical University, Chongqing, China; ^3^Cholestatic Liver Diseases Center of the Institute of Digestive Disease, First Affiliated of Army Medical University, Chongqing, China; ^4^Military Burn Center, The 990th (159th) Hospital of the People’s Liberation Army, Zhumadian, China; ^5^Department of Wound Repair, Institute of Wound Repair, The First Affiliated Hospital of South University of Science and Technology (Shenzhen Peoples Hospital), Shenzhen, China

**Keywords:** pleckstrin homology domain-containing protein family member 1, Rab7, nicotinamide adenine dinucleotide, autophagosome–lysosome fusion, heart disease

## Abstract

Induced autophagy is protective against myocardial hypoxia/ischemia (H/I) injury, but evidence regarding the extent of autophagic clearance under H/I and the molecular mechanisms that influence autophagic flux has scarcely been presented. Here, we report that CD38 knockout improved cardiac function and autophagic flux in *CD38^–/–^* mice and *CD38^–/–^* neonatal cardiomyocytes (CMs) under H/I conditions. Mechanistic studies demonstrated that overexpression of CD38 specifically downregulated the expression of Rab7 and its adaptor protein pleckstrin homology domain-containing protein family member 1 (PLEKHM1) through nicotinamide adenine dinucleotide (NAD)-dependent and non-NAD-dependent pathways, respectively. Loss of Rab7/PLEKHM1 impaired the fusion of autophagosomes and lysosomes, resulting in autophagosome accumulation in the myocardium and consequent cardiac dysfunction under H/I conditions. Thus, CD38 mediated autophagic flux blockade and cardiac dysfunction in a Rab7/PLEKHM1-dependent manner. These findings suggest a potential therapeutic strategy involving targeted suppression of CD38 expression.

## Graphical Abstract

CD38 inhibits transcription of the *Plekhm1* gene and Rab7 gene. CD38 mediates autophagic flux blockade and cardiac dysfunction in a Rab7/PLEKHM1-dependent manner.

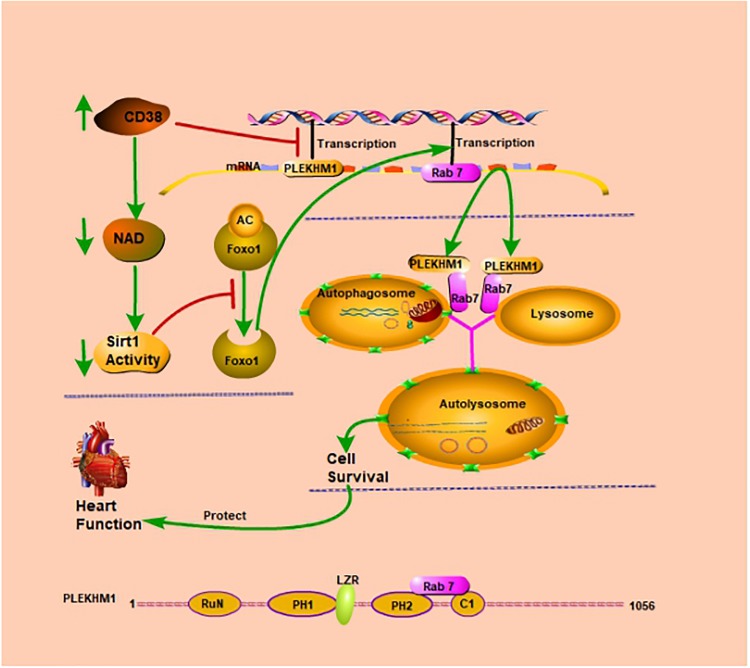

## Introduction

Autophagy is an intracellular lysosomal degradative process that supports cellular homeostasis and survival through quality control of amino acid pools and energy metabolism (Gustafsson and Gottlieb, 2009; Zhang et al., 2018). Macroautophagy involves the segregation of cargo within double-membrane-bound autophagosomes that fuse with and are degraded within lysosomes (Guan et al., 2014). Autophagosome prevalence, commonly regarded as an index of the state of autophagic activation, is determined by the rates of autophagosome formation and clearance. It is therefore a function of flux through the autophagic pathway (Iwai-Kanai et al., 2008). Autophagic activity is increased under many stress conditions, such as starvation, hypoxia, and oxidative stress. Autophagy can enable cells to survive stressors or lead to cell death depending on the context (Gustafsson and Gottlieb, 2008; Zhang et al., 2018). Frequent observations of autophagosomes in dying cells have aroused interest in autophagy as a potential mechanism for a cell death process called type II programmed cell death. However, it is not clear whether the increased abundance of autophagosomes in dying cells reflects upregulation of effective autophagy or impairment of autophagic flux with reduced clearance of accumulated autophagosomes (Klionsky, 2004) followed by secondary activation of programmed cell death (Nishino et al., 2000; Yang et al., 2019).

Hypoxia/ischemia (H/I)-related diseases, such as cardiac dysfunction due to myocardial infarction (MI), tetralogy of Fallot (TOF), stroke, or severe burns, are the most frequent causes of death and disability (Zhu et al., 2010; Zhang et al., 2012; Heusch and Gersh, 2017). Emerging studies have shown that autophagy is a crucial cellular response that degrades incorrectly folded macromolecules and dysfunctional organelles (Wang et al., 2018) and provides bioenergetic intermediates to enable cells to overcome unfavorable stresses. In addition, recent studies have indicated that autophagy is upregulated in response to cardiac H/I injury and is a prominent feature of cardiovascular diseases (Lavandero et al., 2013). However, these studies have mainly focused on the initiation of autophagy; little is known about the degradation of autophagosomes in the myocardium during H/I injury. It is unclear whether autophagosomes fuse with lysosomes and degrade their cytosolic contents in this context (Cui et al., 2017; Hung and Livesey, 2018). Therefore, it is important to investigate the role of efficient autophagic flux and the underlying mechanisms in myocardial H/I injury.

CD38 is a multifunctional protein involved in nicotinamide adenine dinucleotide (NAD) homeostasis and cellular signal transduction (Jin et al., 2007). Under H/I conditions, NAD is usually thought to act as an important survival factor by regulating autophagic flux (Cea et al., 2012; Roest et al., 2018). Previously, CD38 has been implicated to help regulate multiple chronic conditions/diseases, such as aging, obesity, and diabetes, through degradation of NAD (Marchetti et al., 2002; Canto et al., 2012; Camacho-Pereira et al., 2016; Chatterjee et al., 2018). However, the role of CD38 in mediating autophagic flux and H/I-associated cardiomyocyte (CM) death is not yet fully understood. In the present study, we found that the cardiac expression of CD38 was elevated significantly in multiple H/I models. Upregulation of CD38 caused cardiac dysfunction by inhibiting the fusion of autophagosomes and lysosomes under H/I conditions; this effect was mediated by NAD-dependent Rab7 downregulation and non-NAD-dependent PLEKHM1 downregulation.

## Materials and Methods

### Experimental Ethical Approval

Ethical approval to use the human heart samples was obtained from the Research Ethics Committee of Xinqiao Hospital, Chongqing, China, and every patient signed a consent form. Experiments involving animals were performed in accordance with United Kingdom Home Office and European Union guidelines and were approved by the Animal Care Centre of the Third Military Medical University (Army Medical University).

### Generation of CD38-Knockout (*CD38^–/–^*) Mice

*CD38^–/–^* mice were obtained from Prof. Frances E. Lund at the University of Alabama at Birmingham. The strain name of the *CD38^–/–^* mice was B2.129P2-CD38tm1Lnd. The *CD38^–/–^* mice were backcrossed to a C57BL/6J genetic background for more than 10 generations.

### Hypoxia/Ischemia-Related Models

#### Myocardial Biopsy Specimen Collection From Tetralogy of Fallot Patients

Fresh heart tissues were collected from 16 TOF patients with hypoxemia [oxygenation index (OI) ≤ 240; *n* = 8] or normoxemia (OI ≥ 300; *n* = 8) at the Department of Cardiovascular Surgery, Xinqiao Hospital, Chongqing, China. Myocardial samples, mainly from hypertrophic right ventricular myocardium, were collected from patients with TOF undergoing surgery and were divided into two groups depending on the OI upon admission. The ventricular biopsy specimens (100-mg wet weight) were immediately stored in liquid nitrogen. The investigator was blinded to the group allocation during the experimental procedure and when assessing the outcome.

#### Myocardial Infarction Experimental Model

Mice were anesthetized by intraperitoneal injection of amobarbital sodium (66 μg/g of body weight). The animals’ temperature was maintained at a range from 36.5°C to 37.5°C by a heating pad. The chest was opened with a horizontal incision made through the muscle between the ribs (third intercostal space). Ischemia was achieved by ligating the anterior descending branch of the left anterior descending artery (LAD).

#### Thermal Burn Experimental Model

The skin of each mouse was prepared after induction of anesthesia with intraperitoneal amobarbital sodium (66 μg/g of body weight). Then, the dorsum [approximately 25% of the total body surface area (TBSA)] was immersed in 88°C water for 10 s to produce a full-thickness burn injury. After being burned, all burned mice were immediately resuscitated with 1 ml of lactated Ringer’s solution *via* the peritoneum, and water and food were provided *ad libitum*. Experiments were conducted on the mice starting 24 h after the burn challenge.

#### Primary Cardiomyocyte Model of Hypoxia/Ischemia Injury

Neonatal mouse CMs were cultured as described previously (Huang et al., 2015). Neonatal CMs were isolated by enzymatic disassociation from 1-day-old neonatal mouse hearts; the crude method of collagenase type II enzymatic digestion was applied to isolate the cells. The final cell suspension including CMs was separated by allowing the cell suspension to sediment in an uncoated culture dish at 37°C for 1 h. Preplating was performed to help fibroblasts adhere to the culture dish and separate them from the CMs, which remained floating in the suspension. To inhibit fibroblast proliferation, 0.1 mM of bromodeoxyuridine was added. The cells were then used for experiments as stated. After transfection with adenoviruses for 48 h, the cells were subjected to H/I at 37°C under 5% CO_2_ and 1% O_2_ for 12 h. The *in vitro* experiments were conducted 3 times independently.

### Animal and Cell Drug Treatments

Chloroquine (CQ; Sigma-Aldrich, St. Louis, MO, United States) was administered to 12-week-old *CD38^–/–^* mice (*n* = 20; 10 of the mice were in the control group, and the other 10 were in the burn group) daily by intraperitoneal injection at a dose of 5 mg/kg of body weight for 3 days. The last injection was performed 1 h before the burns were produced. Daporinad (FK866, APO866) (Selleck, Houston, TX, United States, s2799) was added to the medium daily to a concentration of 10 μM for 3 days.

### Immunoblot Analysis

Tissue samples were dissected and homogenized in tissue protein extraction reagent (T-PER, Thermo Scientific, Waltham, MA, United States). Cell samples were homogenized in radioimmunoprecipitation assay (RIPA) buffer (Sigma-Aldrich, R0278) with protease inhibitor tablets. The lysates were centrifuged at 16,000 × *g* and 4°C for 15 min to remove insoluble protein. Sodium dodecyl sulfate–polyacrylamide gel electrophoresis (SDS-PAGE) was carried out after equal bicinchoninic acid (BCA)-based protein loading for each sample using gradient gels. The separated proteins were transferred to polyvinylidene difluoride (PVDF) membranes (Millipore, Worcester, MA, United States), blocked with 5% skim milk, and then incubated at 4°C overnight with primary antibodies followed by corresponding secondary antibodies. The specific protein bands were detected using avidin biotinylated horseradish peroxidase with an enhanced chemiluminescence detection kit (GE Healthcare, Pittsburgh, PA, United States). The following antibodies were used in this experiment: anti-CD38 (Santa Cruz Biotechnology, CA, United States, sc-7094), anti-actin (Abcam, Cambridge, United Kingdom, ab5649), anti-Lamin B (Cell Signaling Technology, Danvers, MA, United States, Cat# 13435S), anti-LC3 (Sigma, SAB1306269), anti-P62 (Cell Signaling Technology, Cat# 88588), anti-AMPK (Cell Signaling Technology, Cat# 5832), anti-p-AMPK (Cell Signaling Technology, Cat# 2537), anti-ULK (Cell Signaling Technology, Cat# 8054), anti-p-ULK (Cell Signaling Technology, Cat# 14202), anti-mTOR (Cell Signaling Technology, Cat# 2983), anti-p-mTOR (Cell Signaling Technology, Cat# 5536), anti-P70S6K (Cell Signaling Technology, Cat# 2708), anti-p-P70S6k (Cell Signaling Technology, Cat# 9234), anti-Nampt (Abcam, ab45890), anti-PARP (Cell Signaling Technology, Cat# 9532S), anti-silent mating type information regulation 2 homolog-1 (Sirt1) (Cell Signaling Technology, Cat# 8469S), anti-FOXO1 (Cell Signaling Technology, Cat# 2880S), anti-acetyl (Abcam, ab225500), anti-Rab7 (Abcam, ab50533), anti-Plekhm1 (Biorbyt, orb2306), anti-Atg12 (Santa Cruz Biotechnology, sc271688), anti-caspase3 (Cell Signaling Technology Cat# 9662), anti-cleaved-caspase3 (Cell Signaling Technology, Cat# 9661S), anti-cytochrome *c* (Cell Signaling Technology, Cat# 1140S), anti-VDAC (Cell Signaling Technology, Cat# 4661), and anti-cardiac troponin I (cTnI) (Proteintech Group, Chicago, IL, United States, Cat# 21652-1-AP).

### Electron Microscopy

Left ventricular (LV) myocardium samples or CMs were fixed in 2.5% glutaraldehyde, dehydrated, sliced with a vibratome, recut on a microtome, and stained with uranyl acetate and lead citrate overnight. The sections of heart tissues and CMs were examined by transmission electron microscopy (TEM) (TECNAI 12, Philips, Amsterdam, Netherlands).

### Immunofluorescence

Specimens were fixed in 4% paraformaldehyde for 20 min, permeabilized with or without 0.1% Triton X-100 in phosphate-buffered solution (PBS) for 10 min, and blocked in 3% bovine serum albumin (BSA) for 1 h. Primary antibodies were diluted with PBS, and the coverslips were incubated with the primary antibodies at 4°C overnight. The coverslips were washed in PBS and then incubated with secondary antibodies conjugated with a fluorophore for 1 h at 37°C. The nuclei were then stained for 2 min with 4’,6-diamidino-2-phenylindole (DAPI) (Sigma-Aldrich, 0.5 μg/ml).

### Autophagy Detection Using an mRFP-GFP Adenoviral Vector

Ad-mRFP-GFP-LC3 was purchased from HanBio Technology Co., Ltd. (Shanghai, China); adenoviral infection was performed according to the instructions.

### Gene Expression Analysis

Fresh tissues were removed and immersed in RNAlater^TM^ Solution (Thermo Fisher Scientific, Cat# AM7020). Total RNA was extracted with TRIzol^TM^ reagent (Thermo Fisher Scientific, Cat# 15596018). cDNA was prepared from total RNA using a QuantiNova^TM^ reverse transcription kit (Qiagen, Dusseldorf, Germany, Cat# 205411) according to the manufacturer’s instructions. Quantitative real-time polymerase chain reaction (RT-qPCR) was performed using a QuantiNova^TM^ SYBR^®^ Green PCR kit (Qiagen, Cat# 208054) on an Applied Biosystems^®^ 7500 Real-Time PCR System according to the manufacturer’s instructions. The primers are shown in Supplementary Table S2.

### Apoptosis Assay

An *In Situ* Cell Death Detection Kit (Roche, Basel, Switzerland, Cat# 11684795910) was used to perform an apoptosis assay on deparaffinized LV heart tissue sections. Images of each group obtained under a BX61 Olympus microscope were randomly chosen and further analyzed by Image-Pro Plus 5 software.

### Echocardiography

Mice were anesthetized with a mixture of 1.5% isoflurane and oxygen. Cardiac function was assessed by echocardiography with a Vivid 7 instrument (GE Medical Systems). Images of typical parasternal long-axis, apical four-chamber, and apical five-chamber views were collected. The data acquisition and subsequent analysis were performed with GE Medical Systems software.

### Blood Parameters

Blood samples were collected after removal of eyeballs from anesthetized animals. The serum levels of cardiac troponin T (cTnT) and creatine kinase-MB (CK-MB) were determined using commercial kits.

### Lactate Dehydrogenase Release and Cell Viability Assay

The release of lactate dehydrogenase (LDH) from cells was measured using a CytoTox-ONE^TM^ Homogeneous Membrane Integrity Assay Kit (Promega, Madison, WI, United States, G7890). CM viability was measured by Cell Counting Kit-8 (CCK-8) assay.

### Sirt1 and Sirtuins Activity Measurements

Sirt1 was measured using a SIRT1 Assay Kit (Sigma, CS1040-1KT), and sirtuin activity was measured according to the instructions of a Universal SIRT Activity Assay (Abcam, ab156915).

### Nicotinamide Adenine Dinucleotide and Adenosine Triphosphate Measurements

NAD was measured using an EnzyChrom^TM^ NAD/NADH Assay Kit according to the manufacturer’s protocol (Bioassay Systems, Hayward, CA, United States, #ECND-100), and adenosine triphosphate (ATP) was measured using an ATP kit (Abcam, ab83355).

### Luciferase Reporter Assay

HEK-293T cells were seeded in 48-well plates and cotransfected with the indicated plasmids and luciferase reporters. Forty-eight hours after transfection, the cells were lysed. Then, luciferase activity was measured using a Dual-Luciferase Assay Kit (Promega) on a luminometer (BK-L96C) following the manufacturer’s instructions. Cells were cotransfected with pCDNA3.1 + pGL3-Basic or CD38 + pGL3-Basic plasmids as internal controls. The experiments were performed in triplicate.

### Chromatin Immunoprecipitation

To generate DNA fragments with an average size of 500 bp, neonatal CMs in all groups were crosslinked, lysed, and sonicated. Chromatin Immunoprecipitation (ChIP)-grade antibodies against CD38 (GeneTex, Alton Parkway, Irvine, CA, United States, GTX75086) or control IgG was added to the cell lysate. Aliquots of whole-cell lysate DNA and ChIP-enriched DNA were subjected to PCR using ChIP-specific primers.

### Mitochondria and Nuclear Isolation

Mitochondria were isolated from mouse heart tissues or neonatal CMs, digested with trypsin, homogenized with a glass homogenizer, and then centrifuged at 800 × *g* for 10 min at 4°C. The supernatant was centrifuged at 8,000 × *g* for 10 min at 4°C, and the pellet was retained. The sediment containing mitochondria was washed and centrifuged at 8,000 × *g* for 10 min at 4°C before resuspension. The mitochondrial protein concentration was determined by colorimetry using Quick Start Bradford 1 × dye reagent (Bio-Rad, 500-0250). Nuclei were extracted from minced mouse heart tissue or neonatal CMs using a Nuclear Extraction Kit (Abcam, ab113474) according to the manufacturer’s instructions.

### Mitochondria-Derived Reactive Oxygen Species Measurement

Mitochondria-derived reactive oxygen species (ROS) levels in neonatal CMs were measured using a mitochondrial superoxide indicator (Thermo Fisher Scientific, MitoSOX^TM^ Red, M36008) according to the manufacturer’s instructions.

### Statistical Analysis

The data are presented as the mean ± SEM. Statistical differences between groups were assessed by two-tailed Student’s *t*-test or one-way analysis of variance (ANOVA) with *post hoc* tests, where appropriate. *P* < 0.05 was considered to indicate statistical significance. Statistical analysis was performed with the software program IBM SPSS Statistics, version 22 (SPSS, Inc).

## Results

### CD38 Protein Expression Is Increased in Hearts Subjected to Hypoxia/Ischemia Injury

**FIGURE 1:**
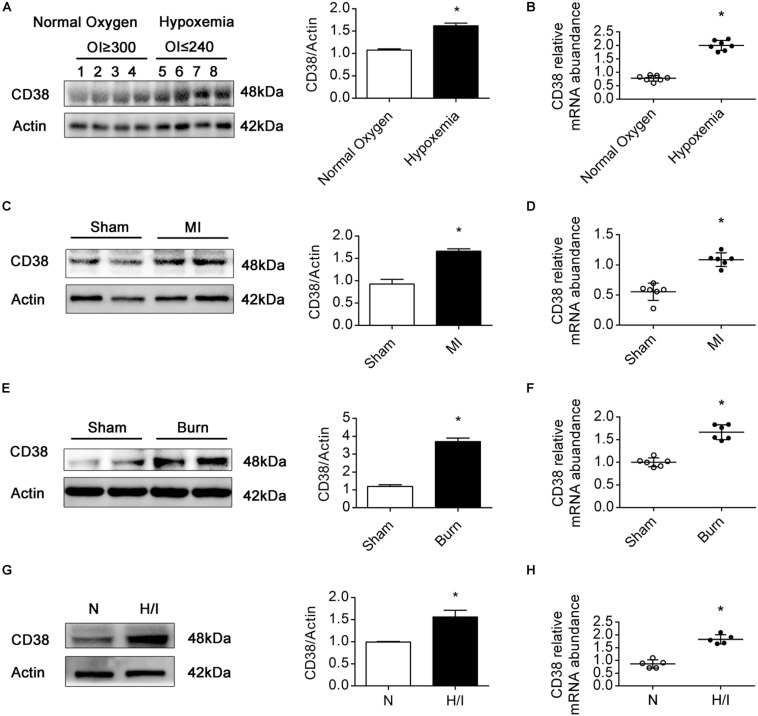
CD38 protein expression is increased in hearts subjected to H/I injury. Representative immunoblot **(A,C,E,G)** and RT-qPCR **(B,D,F,H)** results depicting CD38 in hearts of TOF patients with normoxemia (*n* = 8) and hypoxemia (*n* = 8) **(A,B)**; hearts of mice subjected to sham surgery (*n* = 6) and MI surgery (*n* = 6) **(C,D)**; hearts of sham (*n* = 6) and burned mice (*n* = 6) **(E,F)**; and CMs subjected to H/I (12 h) **(G,H)**. Scale bar, 10 μm. All quantitative results are shown as the means ± SEMs. *P < 0.05. The *P*-values were derived from *t*-tests.

Previous studies have confirmed that patients with TOF, MI, or severe burns suffer from myocardial H/I injury (Huang et al., 2003; Zhu et al., 2010; Anderson and Morrow, 2017). In patients with MI, H/I injury occurs locally, whereas in patients with TOF or severe burns, H/I injury is found in multiple organs, especially in the heart (Supplementary Table S1). To examine the expression of CD38 in the myocardium under H/I conditions, we collected myocardial tissues from TOF patients whose OI values were below 240 or above 300 and found that the protein and mRNA levels of CD38 were obviously higher in patients with hypoxemia (OI ≤ 240) than in patients with normoxemia (OI ≥ 300) (Figures 1A,B). Consistent results were obtained in the comparison between the MI group mice and the sham-operated mice (Figures 1C,D). We also analyzed CD38 expression in myocardial tissues from C57BL/6J mice after severe burn induction or sham surgery. Elevated CD38 mRNA levels and protein expression were observed in the severely burned mice (Figures 1E,F). Furthermore, the upregulation of CD38 after H/I injury was confirmed in cultured CMs using immunoblot analysis and RT-qPCR (Figures 1G,H). These results suggested that the protein content of CD38 was increased in myocardial tissue under multiple types of H/I conditions.

### CD38 Plays a Key Role in Hypoxia/Ischemia-Related Myocardial Injury

**FIGURE 2:**
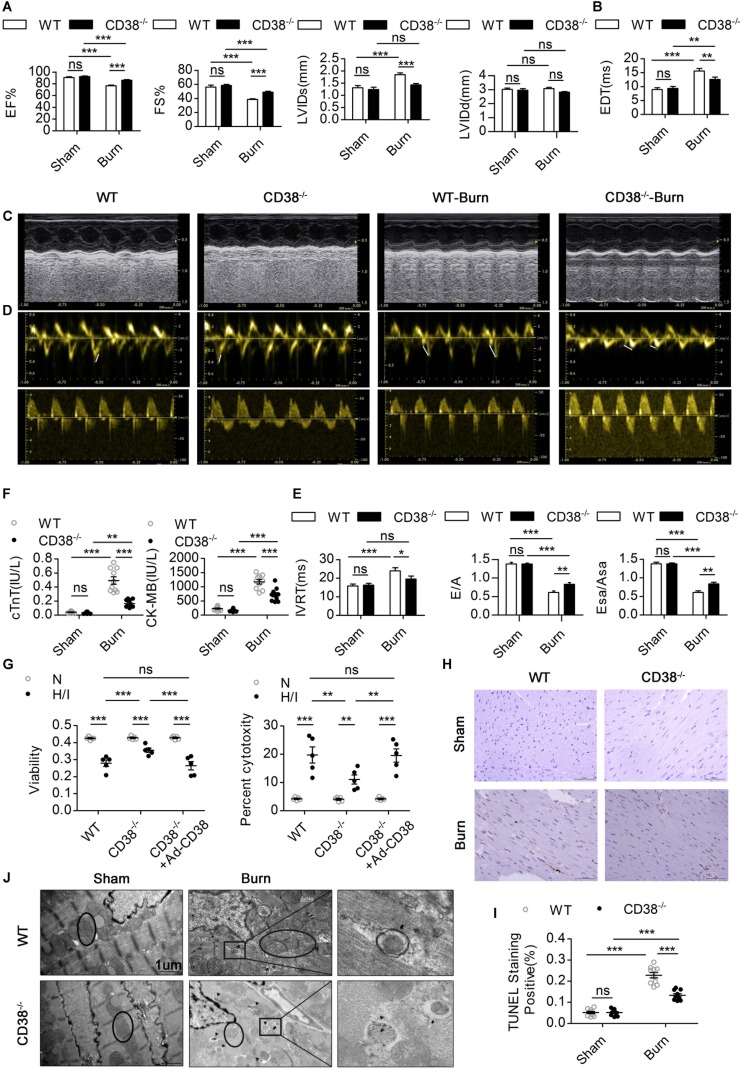
CD38 plays a key role in H/I-related myocardial injury.**(A–E)** The ejection fraction (EF), fractional shortening (FS), LV internal diameter at end-systole (LVIDs), LV internal diameter at end-diastole (LVIDd), deceleration time of mitral inflow (EDT), mitral E velocity relative to mitral A velocity (E/A), peak E wave in earyl diastole at the septal annulus relative to the peak A wave in late diastole at the septal annulus (Esa/Asa), and intraventricular relaxation time (IVRT) are presented (*n* = 10/group). **(F)** Blood markers of cardiac damage [cardiac troponin T (cTnT) and creatine kinase-MB (CK-MB)] (*n* = 10/group). **(G)** Myocardial cell injury under H/I conditions was determined using Cell Counting Kit-8 (CCK-8) assays, and cell viability was determined by LDH release assays. **(H,I)** Representative images and quantitative results depicting TUNEL staining in heart tissues. Scale bar, 50 μm. **(J)** Representative TEM images showing the ultrastructures of LV myofilaments (the black arrows indicate autophagosomes, the black triangles indicate autolysosomes, and the black circles represent mitochondria). Scale bar, 1 μm. The data are shown as the means ± SEMs. *P < 0.05, **P < 0.01, ***P < 0.001, ns, not statistically significant. The *P*-values were derived from one-way ANOVA with Bonferroni’s posttest.

To confirm whether CD38 overexpression is associated with cardiac dysfunction after H/I injury, we subjected 12-week-old male wild-type (WT) and *CD38^–/–^* mice (Supplementary Figure S1A) (20–25 g) to sham surgery or 25% TBSA III° burn injury (Supplementary Figure S1D). The mice were used for experiments 24 h after the procedure. Systolic and diastolic cardiac function and myocardial enzyme levels were comparable between *CD38^–/–^* mice and WT mice. However, *CD38* deficiency significantly inhibited myocardial enzyme release after severe burn injury (Figure 2F) and improved both systolic and diastolic cardiac function (Figures 2A–E). The numbers of TUNEL-positive cells were lower in H/I-subjected hearts from *CD38^–/–^* mice than in those from WT mice (Figures 2H,I). Consistently, compared with WT CMs, *CD38^–/–^* CMs showed reduced release of LDH and increased viability under H/I conditions (Figure 2G). As expected, the protective effect was lost when CD38 was upregulated with Ad-CD38 (Supplementary Figures S1B,C) in *CD38^–/–^* CMs.

To further illustrate how elevated CD38 expression leads to myocardial damage, we examined the ultrastructure of myocardial tissue by TEM. Compared with those of WT burned mice, the hearts of *CD38^–/–^* burned mice exhibited improved myofilamental and mitochondrial structures (Figure 2J). Surprisingly, more autophagosome accumulation and fewer autophagolysosomes were found in the myocardial tissue of WT burned mice than in that of *CD38^–/–^* burned mice (Figure 2J), which suggested that overexpression of CD38 hampered the fusion of autophagosomes and lysosomes.

### CD38 Participates in Hypoxia/Ischemia-Related Myocardial Injury Though Blockade of Autophagic Flux

**FIGURE 3:**
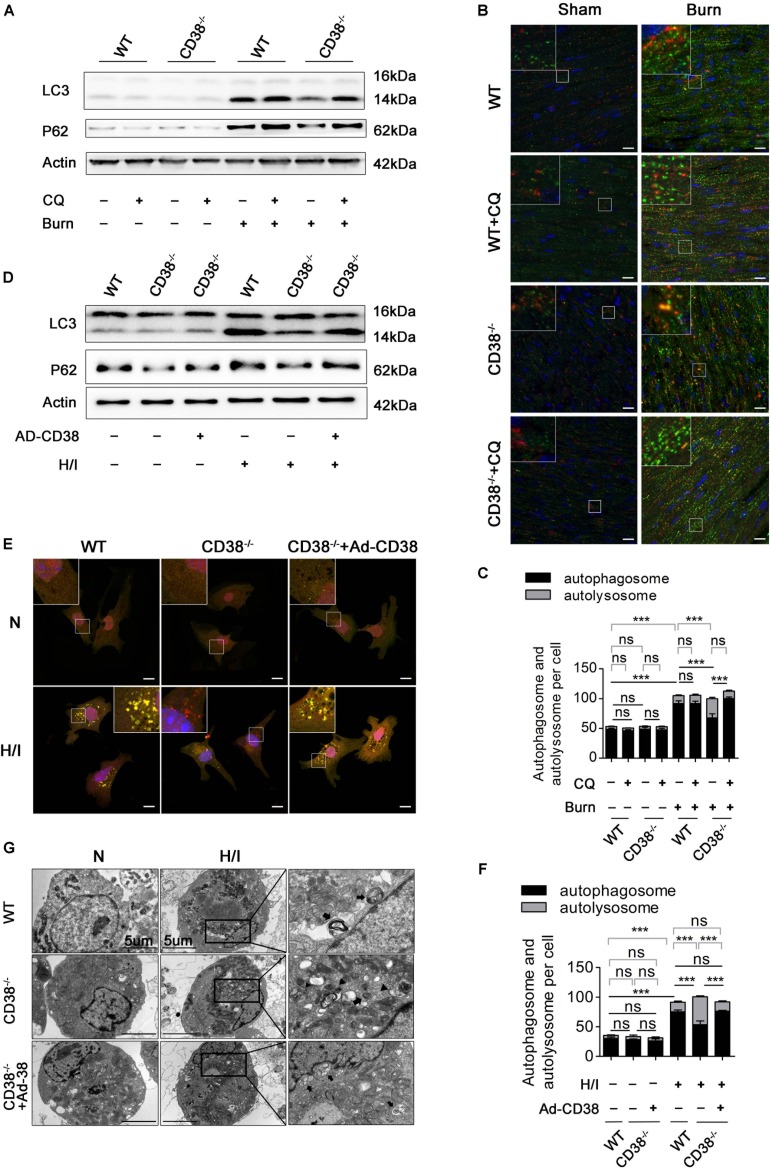
CD38 participates in H/I-related myocardial injury though blockade of autophagic flux. Wild-type (WT) and *CD38^–/–^* mice were subjected to burn injury (24 h) with or without intraperitoneal chloroquine injection (5 mg/kg) (*n* = 10/group). **(A)** Representative immunoblots of LC3 and P62 in whole-cell heart homogenates. **(B)** Representative confocal images of LC3 puncta (green dots) and Lamp1 puncta (red dots) in the heart tissues of adult mice. Scale bar, 10 μm. **(C)** Graph with error bars showing the mean numbers of autophagosomes (green dots) and autolysosomes (yellow dots). **(D–G)** CMs were transfected with an Ad-CD38 adenovirus and then subjected to H/I (12 h). **(D)** Immunoblots showing the expression of LC3, P62, and actin. **(E)** Representative confocal images of mRFP-GFP-LC3 puncta in CMs. Scale bar, 10 μm. **(F)** Bar graph showing the mean numbers of autophagosomes (yellow dots) and autolysosomes (red dots) per cell. **(G)** Autophagosomes and autolysosomes in CMs were observed by TEM (the black arrows represent autophagosomes, and the black triangles represent autolysosomes). Scale bar, 5 μm. The data are shown as the mean ± SEM. ***P < 0.001, ns, not statistically significant. The *P*-values were derived from one-way ANOVA with Bonferroni’s posttest.

**FIGURE 4:**
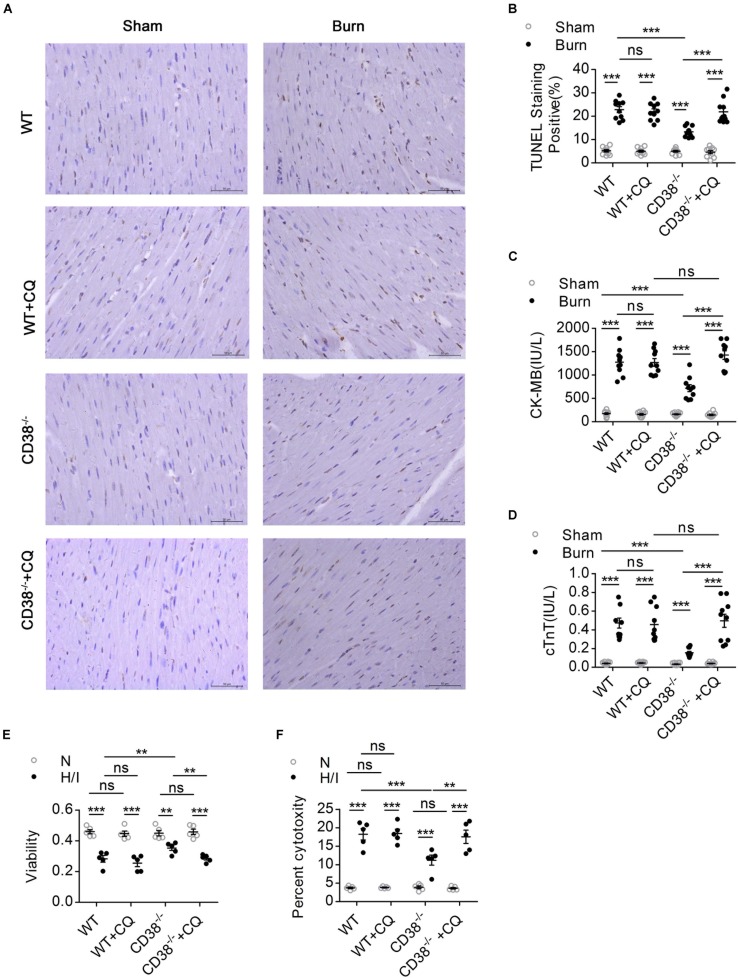
The protective effect of CD38 deficiency on heart function disappeared after blockade of autophagic flux. Wild-type (WT) and *CD38^–/–^* mice were subjected to burn injury (24 h) with or without intraperitoneal chloroquine injection (5 mg/kg) (*n* = 10/group). **(A,B)** Representative images and quantitative results of TUNEL staining for heart tissue apoptosis. Scale bar, 50 μm. **(C,D)** Blood markers of cardiac damage [cardiac troponin T (cTnT) and creatine kinase-MB (CK-MB)] in three mouse models (sham: *n* = 10/group, burned: *n* = 10/group). **(E,F)** Cell viability was determined by using Cell Counting Kit-8 (CCK-8) assays, and LDH release assays were used to determine myocardial cell injury under H/I conditions. The data are shown as the means ± SEMs. **P < 0.01, ***P < 0.001, ns, not statistically significant. The *P*-values were derived from one-way ANOVA with Bonferroni’s posttest.

Previous studies have shown that unobstructed autophagic flux eliminates dysfunctional organelles and provides energy in the heart in response to pathological stresses (Dutta et al., 2013). Thus, we hypothesized that CD38 overexpression would be detrimental to cardiac function under H/I conditions owing to inhibition of autophagic flux. To test this hypothesis, we first examined the mRNA levels of light chain 3-II (LC3-II) and P62 and the activation of the AMP-activated protein kinase (AMPK)/mechanistic/mammalian target of rapamycin (mTOR) signaling pathway, which is involved in the initiation of autophagy. We found obvious increases in mTOR/P70S6K dephosphorylation, AMPK/ULK1 phosphorylation, and LC3-II and P62 mRNA expression that were unrelated to CD38 in both H/I-induced CMs and heart tissues of burned mice (Supplementary Figure S2 and Supplementary Figures S3A,B). However, the increases in LC3-II and P62 protein levels that occurred after H/I or burn treatment were suppressed by knockdown of *CD38* (Figure 3D and Supplementary Figures S2A,B, S3C,D). These results suggested that overexpression of CD38 likely blocked the fusion of autophagosomes and lysosomes rather than inhibited autophagy initiation. Furthermore, CQ, an autophagosome–lysosome fusion inhibitor, was injected into *CD38^–/–^* mice before burn induction. The protein levels of LC3-II and P62 in *CD38^–/–^* burned mice with CQ injection increased to levels close to those in WT burned mice (Figure 3A and Supplementary Figure S3C). Then, we assessed the colocalization of Lamp1 (red) with LC3 (green) by immunofluorescence in myocardial tissue. Deficiency of *CD38* promoted the colocalization of Lamp1 (red) with LC3 (green), but this effect was completely abrogated by CQ treatment (Figures 3B,C). Autophagic flux was also evaluated with Ad-mRFP-GFP-LC3 in CMs. The results showed that CD38 overexpression increased the numbers of GFP/RFP double-positive autophagosomes (yellow) and decreased the numbers of RFP-positive autolysosomes (red) under H/I conditions (Figures 3E,F). Moreover, the TEM results were consistent with the immunofluorescence results (Figure 3G). Functionally, the CD38 deficiency-mediated protection of the myocardium was abolished by blockade of autophagic flux using CQ (Figures 4A–F and Table 1). Thus, CD38 was involved in H/I-related myocardial injury though blockade of autophagic flux.

**TABLE 1:** *In vivo* cardiac function of WT, CD38KO, WT treated with CQ mice, CD38KO treated with CQ mice.

		Sham(*n* = 10/10/10/10)				Burn(*n* = 10/10/10/10)		
				
	WT	WT + CQ	CD38KO	CD38KO + CQ	WT	WT + CQ	CD38KO	CD38KO + CQ
IVSs(mm)	1.26 ± 0.05	1.25 ± 0.06	1.27 ± 0.03	1.26 ± 0.10	1.04 ± 0.05*	0.99 ± 0.05	1.46 ± 0.04^#∧&^	1.08 ± 0.07
IVSd(mm)	0.99 ± 0.03	0.99 ± 0.03	0.90 ± 0.04	0.92 ± 0.04	0.92 ± 0.03	0.83 ± 0.04	0.98 ± 0.04	0.92 ± 0.03
LVIDs(mm)	1.16 ± 0.14	1.16 ± 0.14	1.20 ± 0.05	1.24 ± 0.08	1.85 ± 0.06*	1.79 ± 0.08	1.43 ± 0.06^#∧&^	1.76 ± 0.10
LVIDd(mm)	2.37 ± 0.30	2.37 ± 0.30	2.88 ± 0.05	2.98 ± 0.10	2.98 ± 0.08*	3.04x ± 0.09	2.85 ± 0.06^#∧&^	3.36 ± 0.10
LVPWs(mm)	1.37 ± 0.09	1.37 ± 0.09	1.41 ± 0.07	1.43 ± 0.08	1.16 ± 0.03	1.17 ± 0.03	1.37 ± 0.08	1.14 ± 0.08
LVPWd(mm)	0.93 ± 0.06	0.93 ± 0.06	0.83 ± 0.06	0.84 ± 0.06	0.91 ± 0.03	0.84 ± 0.03	0.92 ± 0.08	0.85 ± 0.02
EF(%)	90.30 ± 1.41	90.30 ± 1.41	92.60 ± 0.64	92.03 ± 1.39	75.10 ± 1.05*	79.50 ± 1.70	86.20 ± 1.07^#∧&^	74.43 ± 1.68
FS(%)	55.10 ± 2.20	55.10 ± 2.20	58.40 ± 1.24	58.30 ± 2.48	37.30 ± 0.92*	41.50 ± 1.80	48.70 ± 1.54^#∧&^	37.67 ± 1.78
EDT(ms)	9.50 ± 0.78	9.50 ± 0.78	9.50 ± 0.67	9.10 ± 0.69	14.18 ± 0.85*	15.00 ± 0.94	10.86 ± 0.90*^∧&^	13.13 ± 0.80
Esa/Asa	1.47 ± 0.05	1.47 ± 0.05	1.38 ± 0.03	1.27 ± 0.05	0.55 ± 0.04*	0.74 ± 0.09	0.76 ± 0.04^#∧&^	0.59 ± 0.03
E/A	1.37 ± 0.04	1.34 ± 0.04	1.37 ± 0.02	1.35 ± 0.02	0.61 ± 0.04*	0.59 ± 0.06	0.83 ± 0.05^#∧&^	0.64 ± 0.04
IVRT(ms)	16.25 ± 0.98	16.25 ± 0.98	17.25 ± 1.00	15.50 ± 1.05	22.25 ± 1.45*	22.79 ± 2.11	16.77 ± 1.18^#∧&^	22.23 ± 1.71
Tei index	0.41 ± 0.04	0.41 ± 0.04	0.43 ± 0.03	0.48 ± 0.04	0.66 ± 0.06*	0.53 ± 0.06	0.41 ± 0.03^∧&^	0.64 ± 0.02
ET(ms)	74.8 ± 2.68	74.8 ± 2.68	70.9 ± 2.41	74.86 ± 2.17	78.40 ± 2.54	78.00 ± 2.94	76.60 ± 1.98	77.56 ± 2.64

EF, ejection traction; EDT, deceleration time of mitral inflow E; E/A, mitral E velocity relative to mitral A velocity; Esa Asa, peak E wave of early diastolic at septal annulus relative to peak A wave of late diastolic septal annulus; ET, ejection time; FS, fractional shortening; IVSs, inten ventricular septal diameter at systole; IVSd, intenventricular septal diameter at diastole; IVRT, intraventricular relaxation time; IVCT, intraventricular contraction time; LVPWs, LV posterior wall thickness at systole; LVPWd, LV posterior wall thickness at diastole; LVIDs, LV internal diameter at systole; LVIDd, LV internal diameter at diastole; LVMM, LV mass. Data are shown as the meaniSEM (^#^*P* < 0.05 vs. the CD38KO Sham group, *P < 0.01, ^@^*P* < 0.001 vs. the corresponding CD38KO Burn group, ^∧^*P* < 0.05 vs. the WT Burn group, ^&^*P* < 0.05 vs. the CD38KO + CQ Burn group). *P*-values were derived from one-way ANOVA with Bonferroni’s post-test.

### Downregulation of CD38 Is Required for Increased Expression of Autophagosome–Lysosome Fusion-Related Genes

**FIGURE 5:**
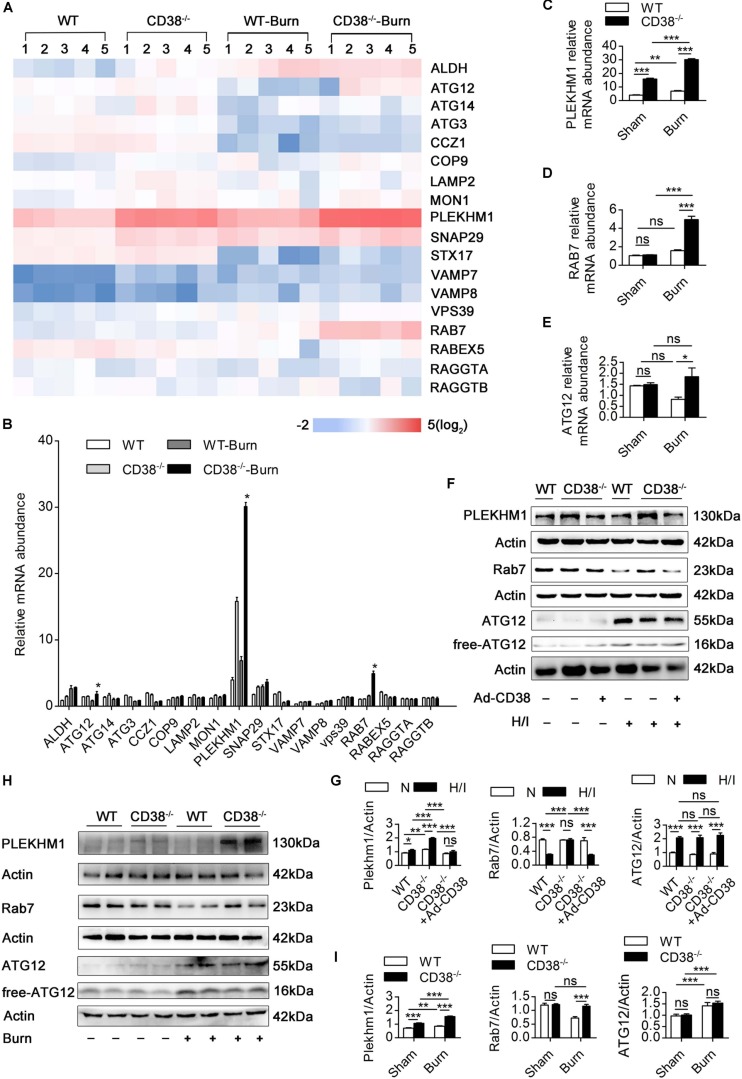
Downregulation of CD38 is required for increased expression of autophagosome–lysosome fusion-related genes. **(A–E)** The expression of various autophagosome–lysosome fusion-associated genes was measured by RT-qPCR. **(F,G)** Comparisons of the indicated parameters between wild-type (WT) and *CD38^–/–^* mice subjected to H/I for 12 h with or without Ad-CD38 adenovirus transfection. Representative immunoblots and quantitative results for PLEKHM1 and Rab7. **(H,I)** Representative immunoblots and quantitative results for PLEKHM1 and Rab7 in two types of mice suffering from burn injury. The data are shown as the means ± SEMs. *P < 0.05, **P < 0.01, ***P < 0.001, ns, not statistically significant. The *P*-values were derived from one-way ANOVA with Bonferroni’s posttest.

Because lysosomal acidification and autophagosome–lysosome fusion-related gene expression are two key factors leading to autophagosome–lysosomal fusion (Godar et al., 2015; Wang et al., 2016), we first examined the effects of CD38 overexpression on autophagosome–lysosome fusion *via* alterations in lysosomal acidification. Lysosomal pH in CMs was determined by staining with LysoSensor (green) and LysoTracker (red). H/I-induced *CD38^–/–^* CMs exhibited a yellow fluorescence similar to that of H/I-induced WT CMs, which indicated that CD38 overexpression did not impair lysosomal acidification (Supplementary Figures S4A,B). We next evaluated the effects of CD38 on autophagosome–lysosome fusion-related gene expression in myocardial tissue by RT-qPCR (Figures 5A,B). *Rab7* and *Plekhm1* were screened as differentially expressed genes associated with CD38 knockout. Interestingly, our results showed that dramatic increases in Rab7 mRNA levels compared with those in WT mice were observed only in *CD38^–/–^* burned mice (Figure 5D), whereas PLEKHM1 mRNA was found to be significantly elevated above WT levels in both *CD38^–/–^* burned mice and *CD38^–/–^* sham mice (Figure 5C). The protein abundance of Rab7 and PLEKHM1 showed similar trends in all the mice (Figures 5F,G). Furthermore, we found that *CD38* knockout-mediated upregulation of Rab7 and PLEKHM1 was attenuated by transduction of *CD38^–/–^* CMs with the Ad-CD38 adenovirus (Figures 5H,I). Notably, the mRNA levels of Atg12 were also significantly higher in *CD38^–/–^* mice post burn than in WT mice post burn (Figure 5E). However, the protein abundance of Atg12 was not affected by CD38 knockout (Figures 5F–I). These results indicated that CD38 participated in the fusion of autophagosomes and lysosomes mainly by regulating the expression of *Rab7* and *Plekhm1*.

### CD38 Mediates Impairment of Autophagosome–Lysosome Fusion and Damage to Cardiomyocytes Through PLEKHM1 and Rab7

**FIGURE 6:**
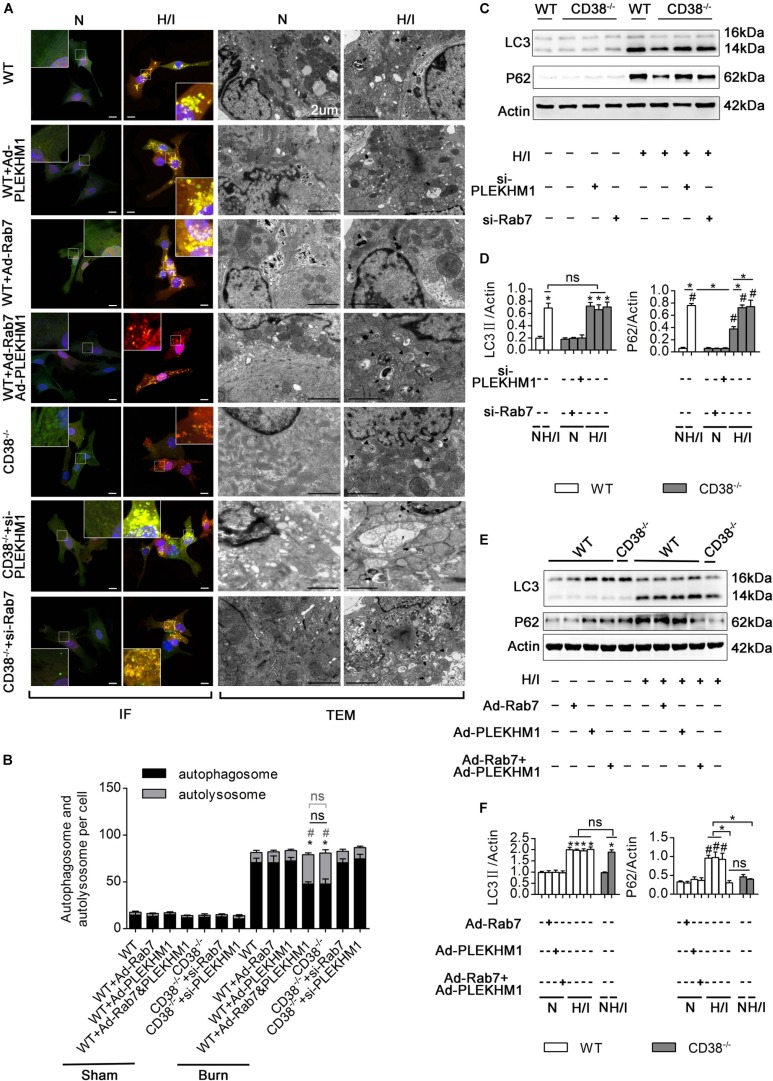
CD38 mediates autophagosome–lysosome fusion through PLEKHM1 and Rab7. **(A–F)** Isolated primary wild-type (WT) neonatal CMs were transfected with an Ad-Rab7 or Ad-PLEKHM1 adenovirus, and primary *CD38^–/–^* neonatal CMs were transfected with a siRNA-Rab7 or si-PLEKHM1 adenovirus. The primary cells were subjected or not subjected to H/I. **(A)** Representative confocal images of mRFP-GFP-LC3 puncta in CMs. Scale bar, 10 μm. CM autophagosomes and autolysosomes were observed by TEM (the black arrows represent autophagosomes, and the black triangles represent autolysosomes). Scale bar, 2 μm. **(B)** Bar graph indicating the mean numbers of autophagosomes (yellow) and autolysosomes (red) per cell. **(C,D)** The influence of Rab7 and PLEKHM1 on the expression of LC3 and P62 in WT neonatal CMs was detected by immunoblotting. Scale bar, 10 μm. **(E,F)** Representative immunoblot results showing the expression of LC3 and P62 in WT neonatal CMs transfected with the siRNA-*Rab7* or si-*Plekhm1* adenovirus. The data are shown as the means ± SEMs. *P < 0.05, ^#^*P* < 0.001 vs. sham, ns, not statistically significant. The *P*-values were derived from one-way ANOVA with Bonferroni’s posttest.

To explore the roles of Rab7 and PLEKHM1 in the CD38-mediated impairment of autophagosome–lysosome fusion, we used adenoviruses to overexpress or knock down Rab7 or PLEKHM1 *in vitro* (Supplementary Figures S1E–H, K–N). As expected, knockout of *CD38* promoted the fusion of autophagosomes and lysosomes (Figures 6A,B) and the degradation of LC3-II and P62 (Figures 6C,D), but these effects were abolished when *Rab7* or *Plekhm1* was knocked down in H/I-induced *CD38^–/–^* CMs (Figures 6A,D). The protective effects also disappeared with knockdown of *Rab7* or *Plekhm1* in CMs from *CD38^–/–^* mice (Supplementary Figures S4C–F). We then applied adenoviruses to stimulate the overexpression of Rab7 and PLEKHM1 in H/I-induced WT CMs. Consistently, overexpression of Rab7 and PLEKHM1 together improved autophagosome–lysosome fusion (Figures 6A,B) and promoted LC3-II and P62 degradation (Figures 6E,F); these effects were not observed when *Rab7* or *Plekhm1* was overexpressed alone in H/I-induced WT CMs (Figures 6A,B,E,F). Moreover, only simultaneous overexpression of Rab7 and PLEKHM1 improved cardiac function (Supplementary Figures S4C–F). These data demonstrated that Rab7 and PLEKHM1 participated together in the CD38-mediated autophagosome–lysosome fusion impairment and CM damage under H/I conditions.

### CD38 Overexpression Inhibits *Rab7* Transcription by a Nicotinamide Adenine Dinucleotide-Dependent Mechanism

**FIGURE 7:**
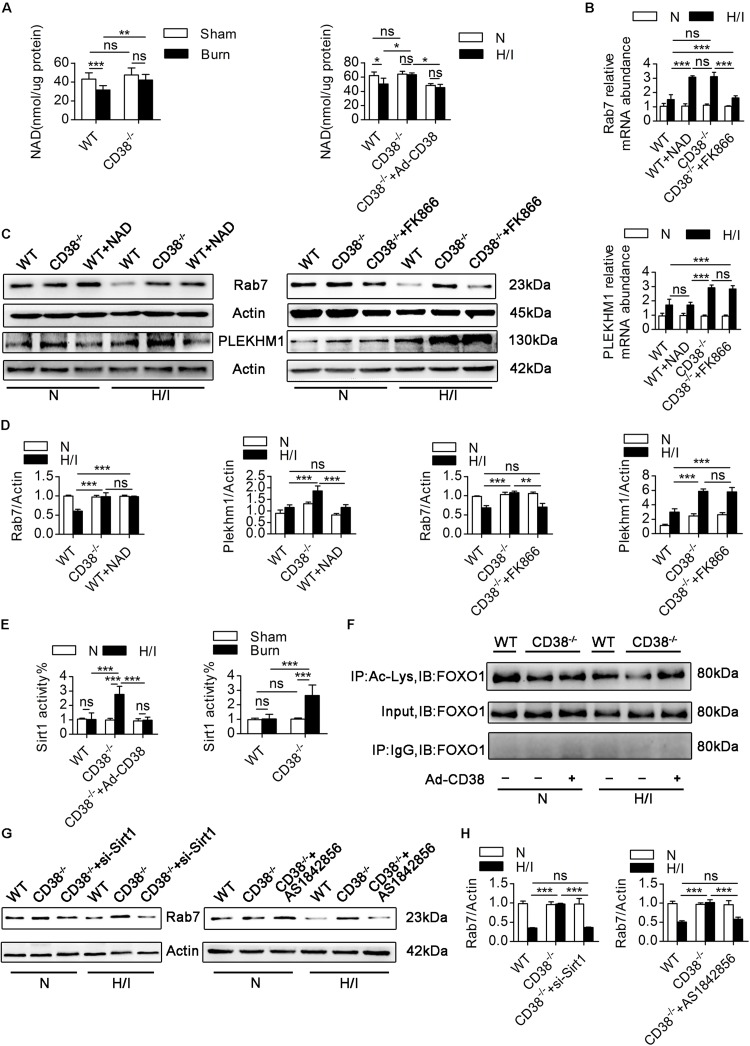
CD38 overexpression regulates Rab7 by a NAD-dependent mechanism. **(A)** The levels of NAD in the heart tissues of adult mice and neonatal CMs were evaluated. **(B)** Relative mRNA expression of Rab7 and PLEKHM1. **(C,D)** Representative immunoblots and quantitative results showing the expression of Rab7 and PLEKHM1. **(E)** Inhibition of CD38 induced Sirt1 activity in the heart tissues of mice after burn treatment and in CMs subjected to H/I. **(F)** FOXO1 was hyperacetylated in response to CD38 knockdown. The lower panel shows representative Western blots of whole-CM homogenate (input) or protein precipitates. The upper panel shows the densitometry results (as the fold changes over the Ctrl value) for acetylated proteins normalized to total proteins. IB, immunoblotting; IP, immunoprecipitation. **(G,H)** The protein expression levels of Rab7 were analyzed by immunoblotting. The data are shown as the means ± SEMs. *P < 0.05, **P < 0.01, ***P < 0.001, ns, not statistically significant. The *P-*values were derived from one-way ANOVA with Bonferroni’s posttest.

CD38 is the main enzyme that metabolizes NAD (Chini et al., 2018), which modulates the acetylation/deacetylation statuses of various transcription factors involved in the regulation of key cellular responses *via* the deacetylase activity of Sirt1 (Zaini et al., 2018). A previous study (Wang et al., 2014) has shown that deacetylation of FOXO1 by Sirt1 plays an essential role in mediating the transcriptional regulation of *Rab7*. Given this information and the observations that CD38 overexpression decreased the mRNA levels of *Rab7* and *Plekhm1*, we hypothesized that a reduction in CD38 expression would promote *Rab7* and *Plekhm1* expression by inhibiting NAD degradation. We first measured the consumption of NAD in myocardial tissue from burned mice. Minimal differences in NAD levels were found between WT and *CD38^–/–^* mice in the sham group. In WT burned mice, NAD levels declined obviously with upregulation of CD38. NAD levels in *CD38^–/–^* burned mice were similar to those in *CD38^–/–^* sham mice (Figure 7A). Consistently, the *in vitro* results showed that *CD38* deficiency maintained NAD levels in CMs subjected to H/I injury; however, this effect was completely abrogated when CD38 was overexpressed using the Ad-CD38 adenovirus in *CD38^–/–^* CMs. It has been reported that reductions in NAD can be caused by increases in the expression of NAD-consuming enzymes (e.g., CD38, PARPs, and Sirtuins) or by decreases in the expression of NAD-synthesizing enzymes (e.g., Nampt). To explore whether upregulation of CD38 was the main factor inducing NAD consumption, we investigated alterations in the abovementioned enzymes *in vivo* and *in vitro*. As shown in Supplementary Figures S5A–D, the protein levels of Nampt, Sirt1, PARP, and cleaved PARP were affected neither by burn injury nor by deletion of *CD38*, and the sirtuins activity was suppressed by overexpression of *CD38* after burn, which indicated that the H/I-mediated NAD reduction was mainly caused by elevated CD38 expression.

We next explored the correlations between the changes in NAD levels and the alterations in Rab7 and PLEKHM1 expression. FK866, a NAD synthesis inhibitor, was used to decrease NAD levels in *CD38^–/–^* CMs, and exogenous NAD was used to increase NAD levels in WT CMs (Supplementary Figure S5E). Exogenous NAD increased the mRNA and protein levels of Rab7 in H/I-induced WT CMs (Figures 7B–D). Consistently, the mRNA and protein levels of Rab7 declined when FK866 was applied to H/I-induced *CD38^–/–^* CMs (Figures 7B–D). However, neither exogenous NAD nor FK866 affected the protein levels of PLEKHM1 (Figures 7B–D). To further test whether CD38 regulates Rab7 though the NAD–Sirt1 axis (Hariharan et al., 2010), we measured the deacetylase activity of Sirt1 *in vivo* and *in vitro*. The results showed that cardiac Sirt1 activity was increased (by nearly 150%) only in heart tissues of *CD38^–/–^* burned mice and *CD38^–/–^* H/I-induced CMs (Figure 7E). FK866 effectively suppressed Sirt1 activity in H/I-treated *CD38^–/–^* CMs, as did adenovirus-mediated CD38 overexpression (Supplementary Figure S5F). Sirt1 in H/I-induced WT CMs was activated only by exogenous NAD treatment (Supplementary Figure S5F). In addition, FOXO1 deacetylation occurred along with the increase in Sirt1 deacetylase activity in response to *CD38* knockdown, and this effect could be reversed by transfection of H/I-induced *CD38^–/–^* CMs with the Ad-siRNA-Sirt1 adenovirus (Figure 7F). Moreover, Rab7 protein expression was higher in *CD38^–/–^* CMs than in WT CMs under H/I conditions, but this difference was abolished when *Sirt1* or FOXO1 was knocked down (Supplementary Figures S1I,J) or inhibited (Figures 7G,H). Together, these results indicated that CD38 overexpression inhibited Rab7 transcription by a NAD-dependent mechanism but that the effect of CD38 on PLEKHM1 expression was not dependent on the NAD pathway.

### CD38 Overexpression Inhibits Transcription of the *Plekhm1* Gene

**FIGURE 8:**
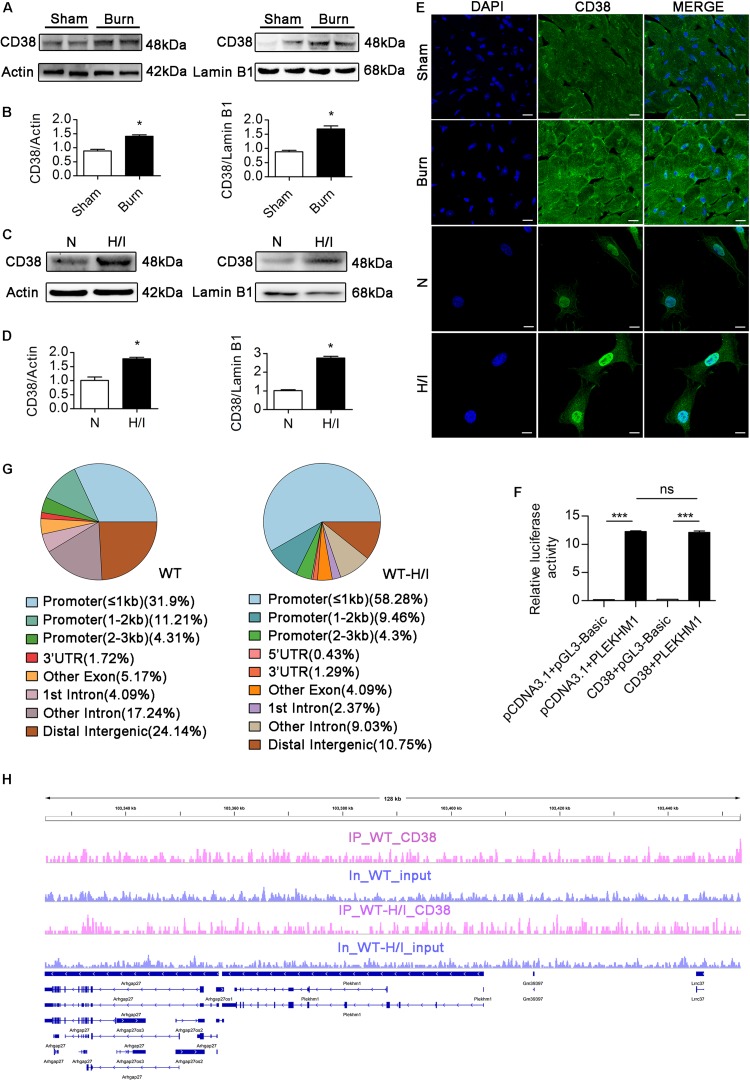
CD38 inhibits transcription of the *Plekhm1* gene. **(A–E)** Immunoblots **(A–D)** and immunofluorescence staining **(E)** of cardiac CD38 protein expression in the cytoplasm and nucleus. Scale bar, 10 μm. **(F)** The dual luciferase reporter system assay preliminarily showed the relationship between CD38 transcriptional activity and PLEKHM1 expression. **(G,H)** ChIP-seq assays confirmed CD38 binding to some related gene promoters. The data are shown as the means ± SEMs. *P < 0.05, ***P < 0.001, ns, not statistically significant. The *P*-values were derived from one-way ANOVA with Bonferroni’s posttest.

Few studies have focused on the transcriptional regulation of *Plekhm1*, and the relationship between CD38 and *Plekhm1* remains elusive. Interestingly, our data showed that there was a significant correlation between CD38 expression and *Plekhm1* expression (Figure 5). Because the mRNA level of *Plekhm1* was decreased by CD38 overexpression, we hypothesized that CD38 could suppress the transcription of *Plekhm1*. To test this hypothesis, we first examined whether CD38 existed in the cell nucleus and whether H/I induced nuclear translocation of CD38. We detected endogenous CD38 expression in the nuclear fractions of CMs, which increased under H/I conditions (Figures 8A–D). We perforated the nuclear membrane with 0.1% Triton X-100 before immunofluorescence staining. As shown in Figure 8E, CD38 could be detected in the cell nucleus, and H/I conditions upregulated both the nuclear and cytomembrane localization of CD38. Then, we performed a luciferase reporter assay and found that CD38 could not bind to the *Plekhm1* promoter sequence (Figure 8F). Previous studies (Shin et al., 2012; Thakore et al., 2015) have shown that some genes can be silenced from a distance. To investigate the possible *Plekhm1-*regulating mechanism of CD38, we employed ChIP-seq assays to comprehensively identify CD38 binding sites on *Plekhm1*. The results showed that CD38 could bind to the promoter sequences of some other transcription factor genes (Supplementary Figure S6), but not the *Plekhm1* gene (Figures 8G,H). Thus, it was likely that CD38 regulated *Plekhm1* by modulating the expression of other transcription factors. The indirect transcriptional regulation of *Plekhm1* by CD38 should be further investigated.

## Discussion

Numerous studies (Imai and Guarente, 2014; Horenstein et al., 2015; Camacho-Pereira et al., 2016) have linked CD38, which modulates cellular NAD homeostasis, to the pathogeneses of multiple conditions, including aging, obesity, diabetes, asthma, and inflammation. NAD is a cofactor for electron transfer during redox reactions and plays crucial roles in cell signaling pathways and in determining the epigenetic status of chromatin (Canto et al., 2015). However, whether CD38 is involved in H/I-induced cardiac dysfunction, the main cause of mortality is currently unknown, although declines in cellular NAD levels have emerged as potential key factors in H/I-related heart disease (Hsu et al., 2009). The purpose of the present study was to explore the role of CD38 in H/I-induced cardiac dysfunction and the underlying molecular mechanism. Our study indicated that overexpression of CD38 accelerated the progression of cardiac dysfunction and worsened outcomes under H/I conditions by blocking autophagic flux through NAD-dependent and NAD-independent pathways.

In the present study, we first demonstrated that CD38 was overexpressed in three types of heart disease with H/I injury and in an H/I-induced cell model. Under H/I conditions, overexpression of cardiac CD38 was generally observed. To further explore the effect of CD38 on cardiac dysfunction, we performed experiments with *CD38^–/–^* mice. Surprisingly, we found that the mice were protected against cardiac dysfunction after burn injury and that this benefit was lost when the mice were treated with CQ, an inhibitor of autophagy that impairs the fusion of autophagosomes and lysosomes. According to previous studies (Liedtke et al., 1976; Camacho-Pereira et al., 2016; Chatterjee et al., 2018), NAD, a substrate of CD38, is an important intermediate in glycolipid metabolism and autophagy, responds to oxidative stress stimulation (Jang et al., 2012), and is essential for maintaining intracellular ATP content. ATP, glycolipid metabolism, autophagy, oxidative stress, and the interactions among them are well known to play roles in the regulation of cardiac function (Yang et al., 2019). However, our data showed that *CD38* knockout and burn treatment did not affect the mRNA levels of various glycolytic, lipolytic, and cardiac oxidative enzymes (Supplementary Figure S7F) or the levels of ATP (Supplementary Figure S7A). Thus, CD38-mediated NAD consumption might not have directly contributed to burn-induced cardiac dysfunction through effects on glucose/lipid metabolism, changes in ATP content, or stimulation of oxidative stress. We also found that exogenous NAD could not effectively improve autophagosome–lysosome fusion (Supplementary Figure S7E), which suggested that NAD played no decisive role in the regulation of autophagic flux. On the other hand, we fortunately found that ablation of *CD38* decreased cardiac mitochondrial damage (Supplementary Figure S7B) and even prevented cardiac mitochondrial apoptosis under H/I conditions and that this benefit was abrogated by CQ treatment (Supplementary Figures S7D,E). These results suggested that unobstructed autophagic flux played an important role in protection against CD38-mediated cardiac dysfunction. Although the mechanism through which CD38 ablation protects the heart from H/I injury requires more exploration, the current study definitively highlights the importance of autophagic flux, rather than NAD, as a critical node linking CD38 to cardiac function under H/I stress.

Autophagosome–lysosome fusion is a highly regulated process that is controlled at the protein level and through the pH of lysosomes (Calcraft et al., 2009; Lavandero et al., 2013; Frudd et al., 2018). In addition, CD38 acts as a potent regulator of autophagic flux by regulating the expression of autophagosome–lysosome fusion-related genes (Hariharan et al., 2010; Qiu et al., 2015) or controlling the pH of lysosomes though NAD (Zhang et al., 2016). The present data showed that CD38 regulated autophagic flux by regulating the core proteins Rab7 and PLEKHM1 rather than by regulating the pH of lysosomes. Consistent with the findings of previous studies (Hariharan et al., 2010; Khan et al., 2015; Qiu et al., 2015), our findings further demonstrated that CD38 regulated Rab7 though the NAD–Sirt1 axis. In our study, the increased Rab7 mRNA levels did not match the protein levels, which suggested the existence of a potent mechanism of H/I-induced Rab7 degradation. This potential mechanism requires further study. In addition, we confirmed that CD38 might act as a transcriptional repressor to indirectly inhibit the transcription of PLEKHM1, and our findings provide direction for further studies exploring the mechanism of PLEKHM1 transcriptional regulation. Although CD38 did not directly repress *Plekhm1* transcription by binding to the *Plekhm1* promoter or other sequences of the gene, CD38 did mediate transcriptional regulation of *Plekhm1* through some other transcription factors. We cannot exclude the possibility that some other proteins are involved in the transcriptional regulation process as cofactors with CD38. The transcriptional regulation of autophagosome–lysosome fusion-related genes should be explored to foster the development of transcriptional strategies that enable promotion of cardiovascular health through *CD38* ablation. It has been proven that PLEKHM1, as an adaptor protein, positively regulates the terminal stages of autophagic pathways through direct interaction with Rab7 and the homotypic fusion and vacuole protein sorting (HOPS) complex (McEwan et al., 2015). Consistently, the aforementioned data obtained in this study strongly suggested that both Rab7 and PLEKHM1 were indispensable for protection against autophagic flux in *CD38^–/–^* burned mice. However, an obvious defect of our current study is that the *CD38^–/–^* mouse model is a systemic knockout model. Although no significant differences in biochemical parameters were noted between the two genotypes of mice, we cannot exclude the potential influences of other tissues and organs on cardiac dysfunction under H/I conditions. Experiments on mice with time- and tissue-specific *CD38* deletion mice may help us solve this problem in the future.

In conclusion, augmentation of CD38 in the heart was sufficient for H/I-induced cardiac dysfunction. Our data revealed an important relationship among CD38, autophagic flux, and cardiac dysfunction under H/I stress. The upregulation of Rab7 and PLEKHM1 expression upon CD38 knockout was deemed an essential mechanism for efficient autophagic flux to prevent the progression of heart dysfunction under H/I conditions. These findings reveal the therapeutic potential of CD38 ablation for amelioration of H/I injury (Graphical Abstract).

## Data Availability Statement

The raw data supporting the conclusions of this article will be made available by the authors, without undue reservation, to any qualified researcher.

## Ethics Statement

The studies involving human participants were reviewed and approved by the Research Ethics Committee of Xinqiao Hospital, Chongqing, China, and experiments involving animals were performed in accordance with United Kingdom Home Office and European Union guidelines and were approved by the Animal Care Centre of the Third Military Medical University (Army Medical University).

## Abbreviations

AMPK: AMP-activated protein kinase
ATP: adenosine triphosphate
CM: cardiomyocyte
CQ: chloroquine
E/A: mitral E velocity relative to mitral A velocity
EDT: deceleration time of mitral inflow E
EF: ejection fraction
Esa/Asa: peak E wave in early diastole at the septal annulus relative to the peak A wave in late diastole at the septal annulus
ET: ejection time
FS: fractional shortening
H/I: hypoxia/ischemia
IVCT: intraventricular contraction time
IVRT: intraventricular relaxation time
IVSd: interventricular septal diameter at diastole
IVSs: interventricular septal diameter at systole
LC3: light chain 3
LDH: lactate dehydrogenase
LVIDd: left ventricular internal diameter at end-diastole
LVIDs: left ventricular internal diameter at end-systole
LVMM: left ventricular muscle mass
LVPWd: left ventricular posterior wall thickness at diastole
LVPWs: left ventricular posterior wall thickness at systole
MI: myocardial infarction
mTOR: mechanistic/mammalian target of rapamycin
NAD: nicotinamide adenine dinucleotide
OI: oxygenation index
PLEKHM1: pleckstrin homology domain-containing protein family member 1
ROS: reactive oxygen species
Sirt1: silent mating type information regulation 2 homolog-1
TBSA: total body surface area
TEM: transmission electron microscopy
TOF: tetralogy of Fallot.
