# Supplementary material for: CD38 Causes Autophagic Flux Inhibition and Cardiac Dysfunction Through a Transcriptional Inhibition Pathway Under Hypoxia/Ischemia Conditions
Source: Front Cell Dev Biol. 2020 Apr 17;8:191. doi: 10.3389/fcell.2020.00191 (PMC7180518; doi:10.3389/fcell.2020.00191)
Supplement: Supplementary file 8 [file Table_1.doc]

**Table S1 Tissue oxygen content of TOF and burn patients**

| **TOF** | | | |  | **BURN** | | | | |
| --- | --- | --- | --- | --- | --- | --- | --- | --- | --- |
| **PID** | **Oxygen flow (L/min)** | **PO_2_ mmHg** | **OI** |  | **PID** | **Scald area (%)** | **Oxygen flow (L/min)** | **PO_2_ mmHg** | **OI** |
|  |
| **1** | 3 | 106 | 321 |  | **1** | 30 | 0 | 103 | 412 |
| **2** | 3 | 115 | 348 |  | **2** | 37 | 0 | 124 | 496 |
| **3** | 3 | 103 | 312 |  | **3** | 35 | 0 | 103 | 412 |
| **4** | 3 | 107 | 324 |  | **4** | 32 | 0 | 100 | 400 |
| **5** | 3 | 105 | 318 |  | **5** | 31 | 0 | 100 | 400 |
| **6** | 3 | 110 | 333 |  | **6** | 35 | 0 | 106 | 424 |
| **7** | 3 | 105 | 318 |  | **7** | 35 | 0 | 153 | 612 |
| **8** | 3 | 101 | 306 |  | **8** | 35 | 0 | 113 | 452 |
| **9** | 3 | 81 | 245 |  | **9** | 56 | 0 | 75 | 300 |
| **10** | 3 | 77 | 233 |  | **10** | 60 | 0 | 40 | 160 |
| **11** | 3 | 59 | 179 |  | **11** | 51 | 0 | 30 | 120 |
| **12** | 3 | 66 | 200 |  | **12** | 54 | 0 | 27 | 108 |
| **13** | 3 | 80 | 242 |  | **13** | 55 | 0 | 36 | 144 |
| **14** | 3 | 71 | 215 |  | **14** | 90 | 0 | 33 | 132 |
| **15** | 3 | 57 | 173 |  | **15** | 95 | 0 | 30 | 120 |
| **16** | 3 | 50 | 152 |  | **16** | 90 | 0 | 33 | 132 |
| **PO_2_,Partial Pressure of Oxygen;OI,Oxygenation Index,PID,Patient's ID** | | | | | | | | | |
