# Supplementary material for: CD38 Causes Autophagic Flux Inhibition and Cardiac Dysfunction Through a Transcriptional Inhibition Pathway Under Hypoxia/Ischemia Conditions
Source: Front Cell Dev Biol. 2020 Apr 17;8:191. doi: 10.3389/fcell.2020.00191 (PMC7180518; doi:10.3389/fcell.2020.00191)
Supplement: Supplementary file 9 [file Table_2.doc]

**Table S2 Primer information**

|  | forward | reverse |
| --- | --- | --- |
| CD38 | 5’-TCTCTAGGAAAGCCCAGATCG-3’ | 5’-GTCCACACCAGGAGTGAGC-3’ |
| ALDH | 5’-AATATCAGTAGCATCGTGAACCG-3’ | 5’-GGAGAGCCCCTTAATCGTGAAA-3’ |
| ATG12 | 5’-CTCAGCCCCACAGCAGTCTTA-3’ | 5’-GTTTCCACTTCCTCAATGCTAGGA-3’ |
| ATG14 | 5’-AGCGGTGATTTCGTCTATTTCG-3’ | 5’-GCTGTTCAATCCTCATCTTGCAT-3’ |
| ATG3 | 5’-ACACGGTGAAGGGAAAGGC-3’ | 5’-TGGTGGACTAAGTGATCTCCAG-3’ |
| CCZ1 | 5’-TGCAGTCATGTGACCTACTTGA-3’ | 5’-AGTCCACTCCAGATAAGTTGGT-3’ |
| COP9 | 5’-GACAACGCCTTCAGTTTCAGA-3’ | 5’-TAGAAGCTGACCGTACACTGG-3’ |
| LAMP2 | 5’-GGGTACTTGCCTTTATGCAGAA-3’ | 5’-CATCGTGTGTCGCCTTGTC-3’ |
| MON1 | 5’-CAGGAGGGTGCGATGTTCG-3’ | 5’-CTAATCTGGCGCATGTCCGTA-3’ |
| PLEKHM1 | 5’-TTGGCCCTCAATGACGGTC-3’ | 5’-TGGCTGACTTGTAGGAGAGTTC-3’ |
| SNAP29 | 5’-TTCGACGATGACGTGGAAGAG-3’ | 5’-GGTACTGCTGCCTGTCAATGG-3’ |
| STX17 | 5’-AAGTATCAGCGGTGCAGAATTT-3’ | 5’-CCTTGACAGGATCTATCATTCGC-3’ |
| VAMP7 | 5’-GACAACTTACGGTTCAAGAGCA-3’ | 5’-TCTCCACGTTGAGCAACTAAATC-3’ |
| VAMP8 | 5’-GGGAGTGCCGGAAATGACC-3’ | 5’-TGAAGTGTTCAGACGTGGCTT-3’ |
| VPS39 | 5’-ATCTGACTGTGGTGCTTAACG-3’ | 5’-ACACTGCAACAATGTATGGAGG-3’ |
| RAB7 | 5’-CCCGAGAACTTCCCTTTTGT-3’ | 5’-TCTTTGTGGCCACTTGTCTG-3’ |
| RABEX5 | 5’-AGACCAATGAGAAAACCCGAAAA-3’ | 5’-TCCTTAGTCACTCGGTCCGTC-3’ |
| RAGGTA | 5’-CACCCTCTGGAACTGTCGC-3’ | 5’-TGCCAAGTGCCATAGGACTTA-3’ |
| RAGGTB | 5’-GTCCTGCATGAACTTTGATGGT-3’ | 5’-CATCTGGTAACTTCTCGGGTCT-3’ |
| LC3 | 5’-GACGGCTTCCTGTACATGGTTT-3’ | 5’-TGGAGTCTTACACAGCCATTGC-3’ |
| P62 | 5’-TGTGGAACATGGAGGGAAGAG-3’ | 5’-TGTGCCTGTGCTGGAACTTTC-3’ |
| CAT | 5’-AGCGACCAGATGAAGCAGTG-3’ | 5’-TCCGCTCTCTGTCAAAGTGTG-3’ |
| COX4 | 5’-TCCCCACTTACGCTGATCG-3’ | 5’-GATGCGGTACAACTGAACTTTCT-3’ |
| Cycs | 5’-GACTACAGCCACGCTTTACCC-3’ | 5’-CCCATCAGCCCAAAACTCC-3’ |
| Gpx4 | 5’-GATGGAGCCCATTCCTGAACC-3’ | 5’-CCCTGTACTTATCCAGGCAGA-3’ |
| Gsr | 5’-ACCGAGGAACTGGAGAATG-3’ | 5’-CAGCATAGACGCCTTTGAC-3’ |
| SOD1 | 5’-AACCAGTTGTGTTGTCAGGAC-3’ | 5’-CCACCATGTTTCTTAGAGTGAGG-3’ |
| SOD2 | 5’-CAGACCTGCCTTACGACTATGG-3’ | 5’-CTCGGTGGCGTTGAGATTGTT-3’ |
| Tomm20 | 5’-GCCCTCTTCATCGGGTACTG-3’ | 5’-ACCAAGCTGTATCTCTTCAAGGA-3’ |
| Txnrd2 | 5’-TTGTGGATGAGCACACAGTTC-3’ | 5’-TTGTGATTCCATATTCCAGGGC-3’ |
| Acadl | 5’-TTT CCG GGA GAG TGT AAG GA-3’ | 5’-ACT TCT CCA GCT TTC TCC CA-3’ |
| Acadrn | 5’-CCAGAGAGGAGATTATCCCCG-3’ | 5’-TACACCCATACGCCAACTCTT-3’ |
| Acadvl | 5’-CCG GTT CTT TGA GGA AGT GAA-3’ | 5’-AGT GTC GTC CTC CAC CTT CTC-3’ |
| Acox1 | 5’-GGG AGT GCT ACG GGT TAC ATG-3’ | 5’-CCG ATA TCC CCA ACA GTG ATG-3’ |
| Acsl1 | 5’-CGCACCCTTCCAACCAACA-3’ | 5’-CGCTATTTCCACTGACTGCAT-3’ |
| Cpt1a | 5’-TGA GTG GCG TCC TCT TTG G-3’ | 5’-CAG CGA GTA GCG CAT AGT CA-3’ |
| Cpt1b | 5’-GGC ACC TCT TCT GCC TTT AC-3’ | 5’-TTT GGG TCA AAC ATG CAG AT-3’ |
| Dgat2 | 5’-GGCTGGCATTTGACTGGAA-3’ | 5’-TGCTGAGATGGTGACCTACGA-3’ |
| Fabp1 | 5’-GGGAAGAAAATCAAACTCACCATC-3’ | 5’-AGTTGTCACCATTTTATTGTCACC-3’ |
| Fabp2 | 5’-TCCCTACAGTCTAGCAGACGG-3’ | 5’-CTCTCGGACAGCAATCAGCTC-3’ |
| LPL | 5’-TCCAGCCAGGATGCAACA-3’ | 5’-CCACGTCTCCGAGTCCTCTCT-3’ |
| PGC-1a | 5’-CCCTGCCATTGTTAAGAC-3’ | 5’-GCTGCTGTTCCTGTTTTC-3’ |
| PGC-1b | 5’-GAGGGCTCCGGCACTTCC-3’ | 5’-CGTACTTGCTTTTCCCAGATG-3’ |
| Plin5 | 5’-AGGGGACTAGACAAATTGG-3’ | 5’-GCTTCTCCGACTTGCC-3’ |
| UCP3 | 5’-TGCTGAGATGGTTGTGTGTCT-3’ | 5’-CCAAAGGCAGAGACAAAGTGA-3’ |
| acetyl-COA carboxylase | 5’-CTCCCGATTCATAATTGGGTCTG-3’ | 5’-TCGACCTTGTTTTACTAGGTGC-3’ |
| 18s | 5’- CCA TCC AAT CGG TAG TAG CG-3’ | 5’-GTA ACC CGT TGA ACC CCA TT-3’ |
