# Supplementary material for: CD38 Causes Autophagic Flux Inhibition and Cardiac Dysfunction Through a Transcriptional Inhibition Pathway Under Hypoxia/Ischemia Conditions
Source: Front Cell Dev Biol. 2020 Apr 17;8:191. doi: 10.3389/fcell.2020.00191 (PMC7180518; doi:10.3389/fcell.2020.00191)
Supplement: Supplementary file 10 [file Presentation_1.pdf]

Supplementary Figure Legends

Fig. S1. Genetic testing, the effect of viral intervention and the degree of burns.

a. Genetic mutations of CD38. **b-c.** Representative Western blot and analysis of identification of Ad-CD38 adenovirus. **d.** Immunohistochemical image showed the full skin scald of mice. **e-h.** Identification of adenovirus of Ad-PLEKHM1 and si-PLEKHM1. **i-j.** Representative Western blot and analysis of identification of sh-sirt1 adenovirus. **k-n.** Identification of Ad-Rab7 adenoviruses and siRNA-Rab7. Data were shown as the mean ± SEM. $^{**}P < 0.01$, $^{***}P < 0.001$, ns, not statistically significant. P values were derived from one-way ANOVA with Bonferroni's post-test.

Fig. S2. CD38 is unnecessary for autophagic induction in the heart.

a-d. Representative immunoblots and analyses of AMPK/m-TOR pathway-related proteins prepared in the cytosolic fractions from heart homogenates. **e-h.** Representative immunoblots of AMPK/m-TOR pathway-related proteins in cardiomyoctes (CMs). Data were shown as the mean ± SEM. $^{*}P < 0.05$, $^{**}P < 0.01$, $^{***}P < 0.001$, ns, not statistically significant. P values were derived from one-way ANOVA with Bonferroni's post-test.

Fig. S3. Effect of CD38 on the mRNA and protein level of LC3-II and P62 .

a-b. Representative RT-qPCR analyses depicting LC3-II and P62 in CMs (a) and the hearts of mice (b). **c-d.** Representative immunoblots analyses of LC3-II and P62 in CMs (c) and the hearts of mice (d) corresponding to figure. 3a and d. Quantitative analyses were shown as the mean ± SEM. $^{*}P < 0.05$, $^{**}P < 0.01$, $^{***}P < 0.001$, ns, not statistically significant. P values were derived from one-way ANOVA with Bonferroni's post-test.

Fig. S4. Effect of CD38 on lysosomal pH and CD38 mediates CMs injury through PLEKHM1 and Rab7.

a-b. WT and *CD38*$^{-/-}$ CMs were stained with LysoSensor (green) and Lysotracker (red) and then analyzed by fluorescence microscopy. **c-d.** Representative images and quantitative analysis showed CMs apoptosis using TUNEL staining. **e-f.** Cell viability was determined using CCK-8 assays, and lactate dehydrogenase (LDH) release assays were used to determine CMs injury under H/I conditions. Data were shown as the mean ± SEM. $^{*}P < 0.05$, $^{***}P < 0.001$,$^{\#}P < 0.01$, ns, not statistically significant. P values were derived from one-way ANOVA with Bonferroni's post-test.

Fig. S5. CD38 regulates the H/I-related cardiac NAD decline and sirt1 activity under H/I conditions.

a-c. Representative immunoblots and analyses of other nicotinamide adenine dinucleotide (NAD)-consuming enzymes. **d.** CD38 altered sirtuins activity, especially Sirt1 activity. **e.** The NAD level regulated by NAD or FK866 in WT and CD38KO mice was measured by a NAD/NADH Quantitation Colorimetric Kit. **f.** Sirt1 activity regulated by the NAD level was measured. Data were shown as the mean ± SEM. $^{*}P < 0.05$, $^{**}P < 0.01$, $^{***}P < 0.001$, ns, not statistically significant. P values were derived from one-way ANOVA with Bonferroni's post-test.

Fig. S6. CD38 has the ability to bind the promoter sequences of some transcription factors.

ChIP-seq assays confirmed CD38 binding to the promoters of some transcriptional factor in WT neonatal cardiomyocytes with or without hypoxic/ischemic (H/I) treatment. Glyceraldehyde 3-phosphate dehydrogenase (GAPDH) served as a negative control.

Fig. S7. CD38 regulates mitochondrial apoptosis through autophagic flux rather than oxidative stress or metabolic disorders.

a. ATP levels were detected and normalized to the percentage in normoxic WT neonatal CMs. **b.** MitoSOX levels were detected by flow cytometry. **c.** Representative confocal images of mRFP-GFP-LC3 punct regulated by NAD level in CMs were shown. **d-e.** Immunoblots analysis revealed the protein levels of caspase-3 (cleaved) and cytochrome C (cytoplasm). **f.** Expression of various glutaminolysis and lipolysis-associated genes and oxidative stress-related genes using RT-qPCR. Data were shown as the mean ± SEM. $^{*}P < 0.05$, $^{**}P < 0.01$, $^{***}P < 0.001$, ns, not statistically significant. P values were derived from one-way ANOVA with Bonferroni's post-test.
